# Association between obesity and miscarriage among women of reproductive age in Nepal

**DOI:** 10.1371/journal.pone.0236435

**Published:** 2020-08-06

**Authors:** Pramesh Raj Ghimire, Blessing J. Akombi-Inyang, Caterina Tannous, Kingsley E. Agho

**Affiliations:** 1 Ujyalo Nepal, Ratnanagar Municipality, Nepal; 2 School of Public Health and Community Medicine, University of New South Wales, Sydney, New South Wales, Australia; 3 School of Health Sciences, Western Sydney University, Penrith, New South Wales, Australia; 4 African Vision Research Institute (AVRI), University of KwaZulu-Natal, Durban, South Africa; University of Mississippi Medical Center, UNITED STATES

## Abstract

**Background:**

Obesity is a major health problem in low and middle income countries (LMICs) and is associated with miscarriage. This study aims to examine the association between obesity and miscarriage among reproductive age women (15–49 years) in Nepal.

**Methods:**

The combined 19160 cross-sectional pregnancy data from the Nepal Demographic and Health Survey (NDHS) for the years 2001, 2006, 2011 and 2016 was utilized. Miscarriage was defined as a spontaneous loss of pregnancy that occurred before the foetus reached 7 months of gestational age. Logistic regression analyses that adjusted for clustering, stratification and sampling weights were used to examine the association between obesity and miscarriage among women of reproductive age.

**Results:**

The odds of miscarriage were 1.45 times higher (Adjusted odds ratio (AOR) =  1.45; 95%Cl: 1.06, 1.98, P = 0.021) among women with obesity. Women who did not use contraception, younger (15–19 years), and older women (35 years or more) were significantly more likely to have miscarriage. Women who smoked tobacco reported higher odds of miscarriage than women who did not smoke tobacco (AOR =  1.27; 95%Cl: 1.07,1.50, P = 0.006). Stratification of maternal smoking status by maternal Body Mass Index (BMI), after adjusting for contraception, mother age and year of survey revealed that tobacco smoking and obesity are associated with miscarriage (AOR =  1.46; 95%Cl: 1.05,2.04, P = 0.025).

**Conclusions:**

Findings from this study show that obesity and tobacco smoking are associated with miscarriage. Smoking cessation, pregnancy planning and counselling on healthy weight for women of reproductive age in Nepal may help promote healthy behaviours and decrease the likelihood of miscarriage.

## Introduction

Miscarriage is a spontaneous loss of pregnancy that occurs before the foetus reaches 7 months of gestational age [1]. Almost 15% clinically recognized pregnancies worldwide end up in miscarriage; with the actual number even higher in developing countries due to unreported miscarriages that often take place at home [2]. Following miscarriage, women experience different forms of psychological morbidity such as anxiety, depression, and post-traumatic stress [3, 4]. In addition, miscarriage adds another layer of vulnerability to women’s future reproductive health. Women with a history of previous miscarriage are more likely to have preterm delivery, postpartum haemorrhage, low birth weight, and perinatal death [3, 5, 6].

While some studies have reported non-modifiable risk factors including maternal age [7–9]; and genetic influences such as chromosomal abnormalities, and abnormal uterus structures [10–14] being associated with miscarriage, other studies have reported modifiable behavioural risk factors as negatively affecting foetal viability. A recent systematic review and meta-analysis of studies conducted in 26 countries found active smoking increases the risk of miscarriage [15]. Similarly, caffeine intake [16] and alcohol use during pregnancy [17, 18] were found to be associated with miscarriage. Three systematic reviews and meta-analyses which examined the association between maternal body mass index (BMI) and miscarriage concluded that being obese [19, 20] and underweight [21] were important predictors of miscarriage. However, these studies were conducted in developed countries. There is no recent study that assess the impact of maternal BMI on miscarriage in low and middle income countries (LMICs). Miscarriage is relatively understudied and there remains little empirical evidence available on the impact of maternal BMI on foetal viability in resource-poor countries such as Nepal.

According to the 2016 Nepal demographic and health survey (NDHS), the proportion of women who were overweight or obese (BMI greater than or equal to 25.0) in Nepal increased from 9% in 2006 to 22% in 2016 [1]. Similarly, the proportion of reported miscarriage over the past 15 years increased by almost two-fold from 4.8% in 2001 to 9.1% in 2016 [1, 22]. Despite this increase in BMI and miscarriage, no study to date has investigated this association.

This study aims to examine the impact of maternal BMI on miscarriage by utilizing pooled data from four NDHS conducted in 2001, 2006, 2011, and 2016. Findings from this study will assist government and non-governmental organizations in the modification of current health policy to improve reproductive health in Nepal thus enabling the country to achieve the Sustainable Development Goal (SDG) Target-3 of ensuring healthy lives and promoting well-being for all at all ages.

## Methods

### Data sources and sample composition

This study used datasets from NDHS 2001 [22], 2006 [23], 2011 [24] and 2016 [1]; and all NDHS were implemented by New ERA with the support of the Ministry of Health of Nepal. The NDHS is a population-based, nationally representative household survey which collects data on maternal and child health, gender, fertility, HIV/AIDS, malaria, family planning and nutrition in LMICs. The NDHS program adopts standardized methods involving uniform questionnaires, manuals, and field procedures to gather information that is comparable across countries. Detailed survey methodology and sampling methods used in gathering the data have been reported elsewhere [1, 22–24]. The NDHS adopted three types of questionnaires, each specifically related to the household, women, and men. These questionnaires were translated into three major languages (Maithali, Bhojpuri, and Nepali), pre-tested, and used to collect information on various demographic and health indicators including a women’s reproductive health outcome such as miscarriage.

**Fig 1:**
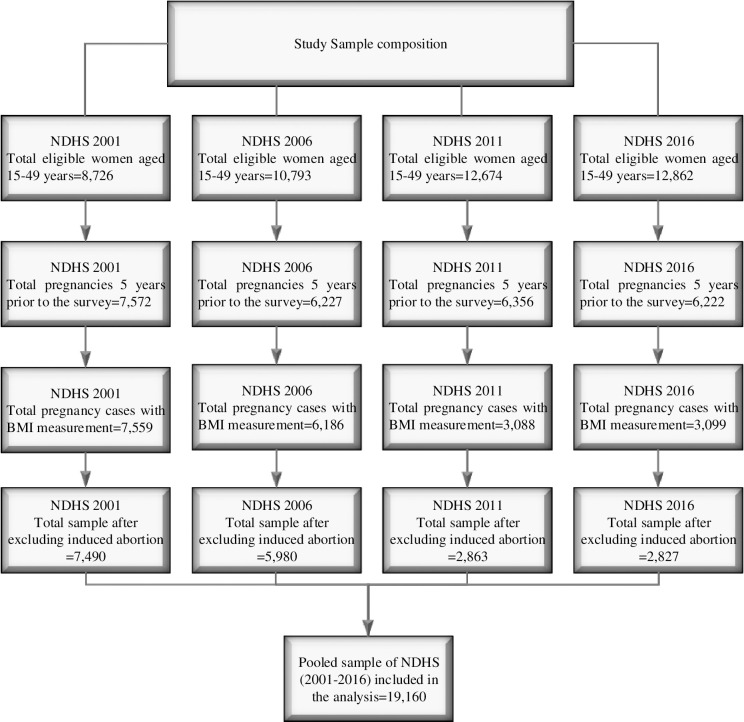
Flow chart for selection of sample from NDHS 2001, 2006, 2011, and 2016.

A total of 45,055 women aged 15–49 years were interviewed across the four NDHS with an average response rate of about 97% [1, 22–24]. Fig 1 shows the flow chart for selection of sample from NDHS 2001, 2006, 2011, and 2016. Reproductive aged women were asked to report all pregnancies that resulted in both live and non-live births. For pregnancies that ended in non-live births, information such as duration of pregnancy and reason of pregnancy termination were collected to identify if the pregnancy ended with a miscarriage or induced abortion. Pregnancies that were intentionally terminated (induced abortion) were excluded. In addition, participants without recorded anthropometric measurements were also excluded from this study.

The analyses were restricted to pregnancies that ended during the 5 years preceding the survey yielding a total weighted sample of 19160. This restriction aimed to minimise recall bias by the mothers, thus strengthening the internal and external validity of this study.

### Outcome variable

Miscarriage was defined as a spontaneous loss of pregnancy that occurred before the foetus reached 7 months of gestational age [1]. Miscarriage was coded as 1 (loss of pregnancy before 7 months of gestational age) and 0 (otherwise).

### Exposure variables

The exposure variables for this study was maternal BMI. In the NDHS (2001–2016), information on maternal weight and height were collected using digital weighing scale and a standard clinical height measurement scale respectively. This information was used to compute maternal BMI. For the purpose of this study, we used the Asian specific BMI cut-offs to categorize maternal BMI and coded as 1 for underweight if BMI <18.5 kg/m2, 2 for normal weight if BMI 18.5–22.99 kg/m2, 3 for overweight if BMI 23–27.49 kg/m2, and 4 for obesity if BMI>27.5 kg/m2 [25, 26].

### Confounding variables

Based on availability of data across all four NDHS (2001–2016), we identified 12 potential confounding variables divided into 4 distinct groups. The time dependent confounding variable was year of survey (survey year 2001, 2006, 2011, and 2016). The community level variables were types of residence (urban and rural), and ecological zone (Mountain, Hill, and Terai). The household level variable was the household wealth index. The household wealth index was constructed using wealth index factor scores available in the respective NDHS data files. For the purpose of this study, the household wealth index was categorised into three groups. The bottom 40%, the next 40%, and the top 20% of households were arbitrarily referred to as poor, middle, and rich households respectively, consistent with previous studies [27, 28]. Individual level variables were maternal education, maternal literacy status, maternal working status, user of contraception, maternal age, and maternal tobacco smoking habit. In the NDHS, women were asked if they have done any work in the last seven days (apart from their own housework). If women replied yes, maternal working status was categorised as working; and if women replied no, maternal working status was categorised as not working.

### Statistical analysis

The characteristics of the study population was described using frequency tabulation. The prevalence and 95% Confidence Interval (CI) of miscarriage for all confounding variables was also estimated. Univariate analyses were performed to examine the independent association between outcome, and all exposure and confounding variables. Multivariate regression models were fitted to examine the impact of maternal BMI on miscarriage. In the univariate analysis, all confounding variables with P value < 0.20 were retained and were used to build a multivariable model [29]. For multivariate regression, a manual backward elimination procedure was applied to remove non-significant variables (P > 0.05). The exposure variable (maternal BMI) was added to all significant confounding variables obtained after elimination processes. The exposure and confounding variables associated with miscarriage at a 5% significance level were reported. Additionally, among women who miscarried, stratified analysis of maternal smoking status by maternal BMI were conducted. All regression analyses were performed by applying the generalized linear latent and mixed models with the log link and binomial family [30] that adjusted for cluster and sampling weights in STATA statistical software, version 14.1 (Stata Corporation, 2017).

## Ethical consideration

The ethics approval for NDHS was obtained from the Ethical Review Board of Nepal Health Research Council and ICF Institutional Review Board. The NDHS datasets are publicly accessible from the DHS website [31]. The first author communicated with MEASURE DHS/ICF International, Rockville, Maryland, USA and was granted permission to download and use the NDHS 2001–2016 datasets. A pre-structured consent statement was read to all study participants and verbal informed consent from each participant (assent on behalf of minors) was recorded by the interviewer.

## Results

A total of 19160 pregnancies were pooled from 2001, 2006, 2011, and 2016 NDHS. From this pooled sample, 1,212 pregnancies resulted in a miscarriage. Table 1 shows the weighted study population, number of miscarriage, miscarriage prevalence and 95% CI of study variables in Nepal. The prevalence of miscarriage among pregnant women increased significantly over these fifteen years from 4.9% (95%CI:4.3–5.6) in 2001 to 10% (95%CI:8.8–11.4) in 2016. Similarly, the prevalence of miscarriage among pregnant women was significantly higher in urban areas, rich households and women not using contraception.

**Table 1:** Weighted study population, number of miscarriage, miscarriage prevalence and 95% CI of study variables Nepal, 2001–2016 (N = 19160).

		Miscarriage	
**Study Variables**	**N**	**n**	**%** (95% CI)
*Confounding variables*			
**Year of survey**			
2001	7490	369	4.9 (4.3, 5.6)
2006	5980	348	5.8 (5.1, 6.6)
2011	2863	212	7.4 (6.1, 8.9)
2016	2827	283	10.0 (8.8, 11.4)
**Type of residence**			
Urban	2991	270	9.0 (7.8, 10.4)
Rural	16169	942	5.8 (5.4, 6.3)
**Ecological zone**			
Terai	9899	606	6.1 (5.5, 6.8)
Hill	7708	504	6.5 (5.9, 7.3)
Mountain	1553	102	6.5 (5.3, 8.0)
**Mother education**			
Secondary or higher	4433	356	8.0 (7.0, 9.2)
Primary	3288	242	7.4 (6.3, 8.5)
No education	11439	614	5.4 (4.9, 5.9)
**Mother's literacy level (N = 19159)**			
read part or whole of the sentence	8742	643	7.4 (6.7, 8.1)
Cannot read	10417	569	5.5 (4.9, 6.1)
**Wealth index**			
Rich	3521	263	7.5 (6.3, 8.8)
Middle	6969	453	6.5 (5.8, 7.2)
Poor	8670	496	5.7 (5.1, 6.4)
**Maternal working status (N = 19159)**			
Not working	5847	426	7.3 (6.4, 8.3)
Working	13312	786	5.9 (5.4, 6.4)
**Mother's current age (years)**			
15–19	1346	136	10.1 (8.2, 12.4)
20–24	6238	354	5.7 (5.0, 6.5)
25–29	5961	339	5.7 (5.0, 6.5)
30–34	3085	184	6.0 (5.0, 7.1)
35–39	1611	110	6.8 (5.4, 8.5)
≥ 40	919	89	9.7 (7.7, 12.1)
**User of contraception**			
Yes	6627	311	4.7 (4.1, 5.4)
No	12533	901	7.2 (6.6, 7.8)
**Maternal tobacco smoking habit**			
No	15476	966	6.7 (5.7, 7.7)
Yes	3684	246	6.2 (5.7, 6.8)
Exposure variable			
**Maternal Body Mass Index**			
Normal	11553	704	6.1 (5.6, 6.6)
Underweight	4419	249	5.6 (4.8, 6.6)
Overweight	2672	204	7.6 (6.5, 8.9)
Obesity	516	55	10.7 (7.6, 14.9)

N = Weighted study population; n = weighted number of miscarriages; CI = Confidence Interval.

### Association of high maternal BMI (obesity) on miscarriage

The univariate analyses revealed that the risk of miscarriage increased significantly among women who were overweight [uOR = 1.25, 95% CI: 1.05, 1.47] and obese (uOR = 1.75, 95% CI: 1.29, 2.36) compared to women with normal weight (Table 2). However, after adjusting for potential confounding variables, obese women were 45% more likely to have a miscarriage (aOR = 1.45, 95% CI: 1.05, 2.04) compared to women of normal weight.

**Table 2:** Unadjusted and adjusted odds ratio and 95% CI for exposure and potential confounding variables associated with miscarriage in Nepal, 2001–2016 (N = 19160).

Exposure Variable	uOR (95% CI)	P-value	aOR (95% CI)	P-value
**Maternal Body Mass Index**				
Normal	Reference		Reference	
Underweight	0.95 (0.81, 1.10)	0.492	0.96 (0.82, 1.12)	0.622
Overweight	1.25 (1.05, 1.47)	0.011	1.13 (0.95, 1.34)	0.18
Obesity	1.75 (1.29, 2.36)	<0.001	1.45(1.06, 1.98)	0.021
***Confounding Variables***				
**Year of survey**				
2001	Reference		Reference	
2006	1.19 (1.01, 1.39)	0.033	1.18 (1.00, 1.38)	0.051
2011	1.55 (1.28, 1.86)	<0.001	1.46 (1.19, 1.78)	<0.001
2016	2.11 (1.75, 2.55)	<0.001	1.81 (1.45, 2.25)	<0.001
**Mother's current age (years)**				
15–19	1.81 (1.47, 2.24)	<0.001	1.73 (1.40, 2.15)	<0.001
20–24	Reference		Reference	
25–29	1.00 (0.85, 1.16)	0.967	1.07 (0.91, 1.25)	0.414
30–34	1.05 (0.87, 1.27)	0.601	1.17 (0.97, 1.43)	0.105
35–39	1.23 (0.98, 1.54)	0.076	1.40 (1.10, 1.78)	0.006
≥ 40	1.87 (1.45, 2.40)	<0.001	2.12 (1.63, 2.77)	<0.001
**Use of contraception**				
Yes	Reference		Reference	
No	1.65 (1.44, 1.89)	<0.001	1.72 (1.50, 1.98)	<0.001
**Maternal tobacco smoking habit**				
No	Reference		Reference	
Yes	1.11 (0.95, 1.30)	0.179	1.27 (1.07, 1.50)	0.006

Model adjusted for: year of survey, types of residence, ecological zone, maternal education, maternal literacy status, wealth index, maternal working status, user of contraception, maternal age, maternal smoking habit, and maternal BMI.

In the multivariate analysis, women who were 15–19 years [aOR = 1.73 (95% CI: 1.40, 2.15)] and ≥ 40 years [aOR = 2.12 (95%CI: 1.63, 2.77)] were more likely to have a miscarriage compared to women who were 20–24 years. Women not using contraception [aOR = 1.72 (95% CI: 1.50, 1.98)] were more predisposed to miscarriage than women using contraceptives. Women who were smokers [aOR = 1.27 (95% CI: 1.07, 1.50)] were more prone to miscarriage than women who were non-smokers.

### Association of tobacco smoking and obesity on miscarriage

Multivariate regression showed that compared to 2001 NDHS, the odds of miscarriage has increased significantly in 2011 NDHS [aOR = 1.46 (95% CI: 1.19, 1.78)], and 2016 NDHS [aOR = 1.81 (95% CI: 1.45, 2.25)]. Women who were tobacco smokers [aOR = 1.27 (95% CI: 1.07, 1.50)] were more prone to miscarriage than women who were non-smokers. Hence, when the analysis was stratified by maternal tobacco smoking habit, the results indicated that obesity was significantly associated with miscarriage especially among women who were tobacco smokers [aOR = 1.46 (95% CI: 1.05, 2.04)] compared to those women who were non-smokers (Table 3).

**Table 3:** Adjusted odds ratio and 95% CI for maternal body mass index on miscarriage by maternal smoking habit in Nepal, 2001–2016 (N = 19160).

Exposure Variable	Miscarriage among smokers		Miscarriage among non-smokers	
Maternal Body Mass Index	aOR (95% CI)	P-value	aOR (95% CI)	P-value
** Normal**	Reference		Reference	
** Underweight**	0.99 (0.83, 1.18)	0.876	0.90 (0.64, 1.27)	0.554
** Overweight**	1.11 (0.92, 1.34)	0.276	1.25 (0.80, 1.94)	0.324
** Obesity**	1.46 (1.05, 2.04)	0.025	1.15(0.40, 3.31)	0.799

aOR = adjusted Odd Ratio; Model adjusted for: year of survey, types of residence, ecological zone, maternal education, maternal literacy status, wealth index, maternal working status, user of contraception, and maternal age.

## Discussion

Obesity has a great impact on female fertility. This could explain the difference observed in gestational loss between obese women and the general population. The present study examines the association between obesity and miscarriage among reproductive aged women (15–49 years) in Nepal. Findings from this study show an increase in the risk of miscarriage with increased BMI among reproductive aged women. The study also reported that smokers who are obese have a greater risk of miscarriage. The double impact of obesity and smoking increases the risk of miscarriage when compared with obese women who are non-smokers or smokers who are of normal weight.

The risk of miscarriage among women of higher BMI (obesity) in this study is in agreement with previous studies conducted in London [32], Sheffield hospital in United Kingdom [33], and North England [34]. Besides increasing the risks of miscarriage, obesity has also been linked to fetal malformation and other adverse pregnancy outcomes [35, 36]. Obesity is reported to compound an array of risk factors which may result in preterm pregnancy loss. For instance, research has shown that obesity is associated with high blood pressure which can complicate pre-eclampsia [34]. Research has also shown that obesity could be a surrogate for pre-gestational and gestational diabetes which is a risk factor for spontaneous abortion [37, 38]. In addition, obesity could also make diabetes harder to manage, thus increasing the risk of complications in the first 13 weeks of pregnancy.

Over the years, Nepal has reported increasing levels of obesity among reproductive aged women [1]. This increasing trend in obesity among women in Nepal is argued to be driven primarily by an obesogenic environment that influences and contributes to an increase in obesity [39]. Urbanization may be one of such factor and this presents a huge reproductive health challenge. The urbanization process has influenced local lifestyle and led to an increase in the uptake of unhealthy diets as well as a decrease in physical activity due to increased reliance on motorized transport [40]. Besides having adverse effects on pregnancy outcomes among reproductive aged women, obesity has also been reported to be associated with increased risks of chronic non-communicable disease (NCD) conditions which are the offshoot of urbanization and modifiable lifestyle factors [41].

Despite the growing level of obesity and its association with miscarriage among women in Nepal, there are still no national health programmes to address this burden. Programmatic health and nutrition interventions aimed at preventing obesity would largely improve reproductive outcomes in women. Such interventions would require a multi-sectoral and multidimensional approach aimed at addressing the social, structural, economic and environmental processes within society which predisposes women to weight gain. Given the increasingly obesogenic environment brought about by rapid urbanization, approaches to prevent miscarriage through obesity management should be aimed at assisting women to change their lifestyles. Therefore, as seen in developed countries, it is recommended that the Nepal government support the development of strategies aimed at modifying the obesogenic environment and lifestyle choices, these could be achieved through appropriate built environment modified for human activities and community-based preconception counselling.

Maternal age is also one of the strongest known risk factors of miscarriage. In this study, maternal age was confirmed as being associated with miscarriage among reproductive aged women in Nepal. The risk of miscarriage was slightly elevated in the youngest mothers and then rose sharply in older mothers. The association between young maternal age and miscarriage could reflect biological mechanisms or indicate unrecognised social causes as well as an effect of reproductive immaturity. Findings from our study is consistent with that of a recent Norwegian study which reported that the proportion of pregnancies that ended in miscarriage was lowest among women between 25–29 years (10 per cent), increased sharply after 30 years, and 53 per cent of pregnancies among women who were 45 or older ended in miscarriage [41]. The observed association between advanced maternal age and miscarriage could be the result of age-related changes such as an increase in conceptions that are chromosomally abnormal or decreasing uterine and hormonal function [42]. A link between increasing maternal age and a higher incidence of chromosomal abnormality has been established in previous studies [43, 44]. However, contradicting inferences have been drawn concerning the possible effect of maternal age on uterus senescence and oocyte quality [45–47]. Also, age might be a surrogate measure of cumulative exposure to unknown factors. Furthermore, findings from this study reveal that lifestyle choices, such as smoking is associated with miscarriage. This is despite other research which has shown that smoking is associated with increased energy expenditure and reduced appetite leading to weight loss [48]. There is consensus that weight loss is protective of miscarriage. In line with this study, the negative association between smoking and miscarriage has been well documented [15, 49, 50]. Research has shown that not only does smoking lead to miscarriage, but the risk of miscarriage also increases with the amount smoked [51]. Hence, there is a need for preconception counselling and public health programs for women of reproductive age to abstain from smoking especially while pregnant.

In this study, non-usage of contraception was associated with miscarriage. Other studies have also reported that women who used oral contraceptives before conception did not show an increased risk of miscarriage [52–54]. The reduction in miscarriage among oral contraceptive users may be due to greater preservation of healthy oocytes, however, this assumption is yet to be confirmed by appropriate laboratory investigations [55]. Contrary to these findings are reports from other studies that contraceptive use could increase chances of miscarriage [56–58]. Oral contraceptives usage can alter the endogenous hormonal milieu, even after discontinuation [59] and cause disruptions in the hormones directly involved in the menstrual cycle. These hormonal disruptions have been found to increase the risk of miscarriage [60–62]. However, the evidence on the association between contraceptive use and miscarriage is inconclusive.

This study was characterised by a number of strengths. First, the findings of this study contribute to the existing body of evidence on the impact of maternal BMI on miscarriage. Second, the dataset used in this study was from population-based nationally representative household surveys with a high response rate (> 90%). The data were pooled to create a large sample size of miscarriage reported within 5 years preceding the surveys. Third, this study applied appropriate statistical adjustments to data obtained from 4 nationally representative surveys and was able to identify the significant factors associated with miscarriage in Nepal to inform policy formulation. This study also had some limitations. First, this study is based on secondary data analysis, and due to the cross-sectional nature of the study design, this paper is unable to establish a causal relationship between obesity and the occurrence of miscarriage. Second, the information on miscarriage is from retrospective data based on self-report from mothers and this could be a potential source of recall and misclassification bias. Third, this study was not able to include important confounders such as the use of caffeine and alcohol which have been previously identified as important modifiable risk factors for miscarriage [16–18]. Fourth, the results should be interpreted with caution while comparing results with non-Asian populations because this study has used BMI for Asian populations. Finally, miscarriage and other pregnancy complications might share underlying causes, which could be biological conditions or unmeasured common risk factors, hence, care should be taken in interpreting and applying the findings of this study.

## Conclusions

This study shows that over the study period (2001–2016) miscarriage has increased significantly in Nepal. Maternal obesity and tobacco smoking are key modifiable risk factors for miscarriage in Nepal. Interventions aimed at maintaining healthy weight and lifestyle choices among women of reproductive age are needed to reduce miscarriage. On an individual level, obesity and the effects of smoking on miscarriage should be incorporated into medical counselling so it can be taken into consideration in decisions prior to conception. In addition, cost-effective community-based health promotion initiatives aimed at educating women on the importance of healthy dietary intake and lifestyle are urgently needed in Nepal to enable the country to achieve the SDG Target-3 of ensuring healthy lives and promoting well-being for all at all ages by 2030.
